# Disordered eating in sexual and gender minority adults: the roles of gender variance, appearance anxiety, and internalized ideals

**DOI:** 10.3389/fpsyg.2025.1658324

**Published:** 2025-10-09

**Authors:** Ana-Sofia Micu, Alin-Sebastian Godeanu, Vasile Constantin

**Affiliations:** Department of Applied Psychology and Psychotherapy, Faculty of Psychology and Educational Sciences, University of Bucharest, Bucharest, Romania

**Keywords:** eating disorders, gender variance, body image, sexual and gender minorities, social appearance

## Abstract

**Introduction:**

Sexual and gender minority (SGM) individuals face elevated risks for disordered eating and body image disturbances, shaped by minority stress, internalized beauty norms, and gendered societal expectations. This study examined (1) whether SGM individuals in Romania report higher levels of disordered eating than cisgender heterosexuals, and (2) whether appearance-related anxiety and internalized beauty ideals mediate the relationship between gender identity/expression and disordered eating within the SGM group.

**Methods:**

A total of 270 Romanian adults (aged 18+), including 225 identifying as SGM, completed self-report measures of disordered eating, gender variance, femininity, appearance-related social anxiety, and thin-ideal internalization. Statistical analyses included Mann–Whitney U tests, bivariate correlations, and percentile bootstrap mediation models.

**Results:**

Sexual and gender minority participants reported significantly higher disordered eating symptoms than cisgender heterosexuals. In the SGM group, social appearance anxiety partially mediated the relationship between gender variance and disordered eating (β = 0.1036, *p* = 0.008), while gender variance showed a significant negative direct effect (β = −0.1465, *p* = 0.009). The indirect effect of femininity through thin-ideal internalization was marginal (β = 0.0852, *p* = 0.051). Both appearance-related anxiety (β = 0.5715, *p* < 0.001) and thin-ideal internalization (β = 0.6549, *p* < 0.001) were robust predictors of disordered eating.

**Discussion:**

Findings underscore the heightened vulnerability of Romanian SGM individuals to disordered eating and illuminate the dual role of gender expression as both a stressor and a potential protective factor. Appearance-related anxiety and internalized ideals emerged as key mechanisms, though with nuanced and partly inconsistent effects. By situating these processes within an under-researched Eastern European context, the study contributes culturally specific insights to the global literature on SGM health disparities. Future research should employ intersectional designs and incorporate broader constructs such as muscularity-oriented dissatisfaction and compensatory behaviors.

## 1 Introduction

Sexual and gender minority (SGM) individuals face a heightened risk of developing disordered eating behaviors and body image disturbances, compared to their cisgender heterosexual counterparts (Nagata et al., 2024). This disparity is substantial: prevalence rates for anorexia nervosa, bulimia nervosa, and binge eating disorder are significantly higher among SGM populations (Nagata et al., 2020). US-based data show particularly elevated risks among transgender individuals, with 10.5% of transgender men and 8.1% of transgender women reporting clinician-diagnosed eating disorders. However, most of these data come from North America and Western Europe. Far less is known about how these risks manifest in countries like Romania, where SGM individuals face different structural barriers, cultural norms, and stigma dynamics (e.g., Brytek-Matera, 2023; Mezza et al., 2024).

This vulnerability arises from a complex interplay of psychosocial and cultural factors, including stigma, minority stress, and the internalization of beauty norms. Adolescence is a particularly sensitive period. For SGM youth, the challenge of forming an identity is compounded by the need to navigate prejudice, rejection, and societal expectations around gender expression and appearance (Roberts et al., 2022). During this stage, appearance becomes a key site of regulation and anxiety, especially when identity and presentation diverge. Many SGM adolescents engage in disordered eating as a way to either suppress or accentuate gendered physical traits in order to “pass” or reduce dysphoria (Romito et al., 2021). These risks persist into adulthood, although they vary across SGM subgroups. For example, lesbian women may be less affected by traditional beauty standards and objectification, potentially offering some protection against eating disorders (Kozee and Tylka, 2006). However, other research suggests no significant differences from heterosexual women, but rather different symptom patterns, such as higher compulsive eating and less restrictive behavior (Dotan et al., 2019). Bisexual women may face additional risks due to unique stressors like objectification, fetishization, and binegativity (Dyar and Feinstein, 2018; Roberts et al., 2022).

Gay and bisexual men consistently report higher levels of body dissatisfaction and disordered eating than heterosexual men (Nagata et al., 2019). They face increased pressure to conform to body ideals within the LGBTQ+ community, where muscularity and leanness are often emphasized. This has led to higher rates of compulsive exercise, restrictive eating, and even body modification (Wood, 2004; Morrison et al., 2004; Frederick and Essayli, 2016). Among SGM men, muscular dysmorphia is a growing concern, with body control strategies focused more on muscularity than thinness (Chaney, 2008). Transgender individuals also show elevated vulnerability to eating disorders, often related to gender dysphoria - the psychological distress caused by the mismatch between gender identity and assigned sex (Ålgars et al., 2012; Jessen et al., 2021). Disordered eating in this context may serve as a form of body control or coping mechanism to modify gendered traits.

Despite this growing body of evidence, the Eastern European region remains largely underrepresented in empirical research on disordered eating among SGM populations. In countries like Romania, where LGBTQ + rights remain contested, public discourse is often hostile, and access to gender-affirming care is severely limited, the mechanisms leading to disordered eating may differ significantly (Brytek-Matera, 2023; Ünsal et al., 2025). Yet few studies have explored how contextual stressors such as conservative gender norms, structural stigma, and limited mental health infrastructure influence these outcomes in SGM individuals living in Romania.

Furthermore, most research and psychometric tools on eating disorders were developed for young, cisgender, white women from higher socioeconomic backgrounds (Murray et al., 2017). As a result, the specific experiences and symptoms of SGM individuals remain underrepresented in both clinical practice and empirical literature. This gap is particularly salient in Eastern Europe, where few studies have examined how minority stress and appearance norms affect SGM individuals (Mezza et al., 2024). Romania offers a compelling case, as factors such as religious conservatism, stigmatizing public discourse around LGBTQ + rights, and underdeveloped mental health infrastructure may amplify these risks (Latu et al., 2024).

To understand these dynamics, researchers have drawn on two primary frameworks: the Minority Stress Model (Meyer, 2003) and Sociocultural Theories. The former explains how chronic exposure to stigma, discrimination, and internalized negativity increases psychological distress in SGM individuals (Testa et al., 2014; Ramirez and Galupo, 2019). The latter focuses on how societal messages transmitted through peers, family, and media establish and enforce idealized body norms (van den Berg et al., 2002; Gill, 2021). Within the LGBTQ+ community, additional pressures may arise from subcultural beauty standards (e.g., “twinks,” “jocks,” “butches”) that create further body-related stress (Jankowski et al., 2014; Fogarty and Walker, 2022).

Understanding these processes within an under-researched Eastern European context such as Romania offers unique insights into how social appearance anxiety and thin-ideal internalization may function as psychosocial risk mechanisms for disordered eating among SGM individuals. By examining these associations, the present study contributes to a more inclusive and context-sensitive understanding of eating disorder vulnerability.

### 1.1 Purpose of the study and hypotheses

Given the limited research on disordered eating among sexual and gender minority (SGM) individuals–particularly in Eastern European contexts–the present study aimed to clarify how gender identity, gender expression, and key psychosocial mechanisms contribute to disordered eating behaviors in a Romanian sample. Specifically, it compared SGM and non-SGM individuals and examined the roles of gender expression, minority status, social appearance anxiety, and thin-ideal internalization in shaping maladaptive eating patterns. Building on prior literature linking gender identity, body image concerns, and sociocultural appearance norms, two mediation models were tested to examine the psychological mechanisms contributing to disordered eating in this understudied population.

Based on these objectives, the following hypotheses were developed:

H1: SGM participants will report significantly higher levels of disordered eating behaviors compared to non-SGM participants.

H2: In the SGM group, social appearance anxiety will be statistically associated with both gender variance and disordered eating behaviors, consistent with a mediational model.

H3: In the SGM group, thin ideal internalization will statistically mediate the association between femininity and disordered eating behaviors.

## 2 Materials and methods

### 2.1 Participants and procedure

This study included 270 Romanian participants (M_age = 24.7 years, SD = 6.22; range: 18–63). Eligibility criteria required participants to be at least 18 years old and residing in Romania at the time of the study. Participants were recruited through convenience sampling between October 2024 and March 2025 using both online and offline strategies designed to ensure diverse representation within the Romanian sexual and gender minority (SGM) community. Online outreach included posts on social media platforms (e.g., Facebook, Instagram), as well as targeted messages shared by individuals with visibility in LGBTQ+ and activist circles. Notably, an environmental and human rights activist with a large Romanian following promoted the study via Instagram. Offline recruitment was conducted in two LGBTQ+ -affirming venues in Bucharest, where printed flyers containing the survey link and QR code were displayed. Members of local drag performance houses were also contacted directly and encouraged to share the study within their networks. Eligibility criteria included being at least 18 years old and residing in Romania at the time of participation. All participants provided informed consent prior to beginning the survey, in compliance with the General Data Protection Regulation [GDPR; Regulation (EU) 2016/679]. Participants were informed about the purpose of the study, the voluntary nature of their participation, and how their data would be processed and stored.

The anonymous survey was administered via Google Forms (Google LLC) and responses were stored securely on a password-protected Google Drive account. According to Google’s published policies, data storage and transfer comply with GDPR cybersecurity and privacy requirements. No compensation (monetary or otherwise) was provided for participation. The study protocol adhered to ethical standards for research involving human participants, including voluntary participation, anonymity, informed consent, and the right to withdraw at any time without penalty.

Of the 271 individuals who accessed the survey, one was excluded due to missing data on gender identity and sexual orientation. The final sample included diverse sexual orientations and gender identities: homosexual (*N* = 94; 34.8%) and bisexual (*N* = 93; 34.4%), followed by heterosexual (*N* = 51; 18.9%). Less frequently reported orientations included pansexual (*N* = 25; 9.3%), queer (*N* = 3; 1.1%), and asexual/demisexual (*N* = 3; 1.1%). One participant (0.4%) reported being in the process of exploring their sexual orientation and identified as questioning.

Regarding gender identity, the most frequently reported identity was cisgender woman (*N* = 156; 57.8%), followed by cisgender man (*N* = 75; 27.8%). Non-binary individuals represented 8.5% of the sample (*N* = 23), and transgender individuals 4.8% (*N* = 13), which included transgender women (*N* = 3; 1.1%) and transgender men (*N* = 10; 3.7%). Less frequently reported gender identities included gender fluid (*N* = 1; 0.4%), agender (*N* = 1; 0.4%), and genderqueer man (*N* = 1; 0.4%).

In terms of sex assigned at birth, 186 participants (68.9%) reported being assigned female at birth, and 84 (31.1%) reported being assigned male. Two participants (0.7%) reported being born with intersex characteristics. The mean age at which they became aware of these characteristics was 8 years. Both identified as cisgender women.

A total of 8 participants (3%) reported undergoing hormonal treatment to feminize or masculinize physical characteristics. One transgender woman reported feminizing hormone therapy, while the remaining seven reported masculinizing therapy and identified either as transgender men (*N* = 6) or non-binary (*N* = 1).

Based on participants’ gender identity, sexual orientation, and intersex status, two mutually exclusive groups were created: the sexual and gender minority (SGM) group and the non-SGM group. The non-SGM group included only cisgender, heterosexual individuals who were not born with intersex traits. The SGM group included all other participants - those identifying as sexual or gender minorities and/or born with intersex characteristics. The SGM group included 225 participants (83.3%), while the non-SGM group included 45 participants (16.7%). The mean age in the SGM group was 24.0 years (SD = 4.47; range: 18–52), with 59.5% having completed higher education. The non-SGM group had a mean age of 28.4 years (SD = 10.9; range: 18–63), and 64.4% reported completed higher education. Within the non-SGM group, 26 participants were cisgender women (*M* = 26.0 years, SD = 9.24; range: 21–63), and 19 were cisgender men (*M* = 27.8 years, SD = 13.0; range: 18–55).

An *a priori* power analysis was conducted using G*Power software (Faul et al., 2009) to determine the minimum sample size required for a Mann–Whitney U test. Assuming a medium effect size (*d* = 0.5), a significance level of α = 0.05, and a statistical power of .95, the recommended sample size was 184 participants.

### 2.2 Instruments

#### 2.2.1 Gender variance scale (GVS)

Yarrow et al. (2023) was used to assess participants’ subjective adherence to feminine or masculine aspects of gender identity across four domains: feelings, appearance, behavior, and interests. Respondents rated their identification with typical expressions of femininity and masculinity on 10 items using a 9-point Likert scale (1 = Not at all; 9 = Completely). Scores were aggregated into two subscales – Femininity and Masculinity – and used to compute the Gender Variance Ratio (GVR), which reflects deviation from gender norms based on sex assigned at birth. Internal consistency in the present study was excellent for both subscales (α = 0.93). In the present study, we used two distinct indicators derived from the GVS: (1) Femininity scores, reflecting participants’ subjective identification with stereotypically feminine traits (e.g., appearance, interests, emotional expression), and (2) the Gender Variance Ratio (GVR), computed as the deviation from culturally expected gender traits based on sex assigned at birth. Although the GVS includes both Femininity and Masculinity subscales, only Femininity was used in the mediation model, due to its stronger theoretical link with thinness-oriented ideals. Prior research suggests that internalization of sociocultural thinness norms is more closely tied to femininity than masculinity (Green et al., 2008). Femininity and GVR were treated as distinct predictors in the mediation models given their different conceptual meanings.

#### 2.2.2 Social appearance anxiety scale (SAAS)

Hart et al. (2008) was employed to measure anxiety regarding negative evaluation of one’s physical appearance. This unidimensional instrument includes 16 items rated on a 5-point Likert scale (1 = Not at all; 5 = Extremely). One item is reverse-coded. The scale demonstrated excellent internal consistency in the current sample (α = 0.96).

#### 2.2.3 Thin ideal internalization assessment (THIINA)

(Kidd et al. (2023) was used to assess the internalization of socio-cultural beauty ideals emphasizing thinness. The scale includes 17 items grouped into three subscales: Thin Idealization (6 items), Thin Overvaluation (8 items), and Thin Behavioral Drive (3 items). Responses were provided on a 5-point Likert scale (1 = Strongly disagree; 5 = Strongly agree). Internal consistency in the current study ranged from good to excellent: Thin Idealization (α = 0.91), Thin Overvaluation (α = 0.89), Thin Behavioral Drive (α = 0.84), and Composite Score (α = 0.94).

#### 2.2.4 Eating attitudes test (EAT-26)

Garner et al. (1982) was administered to screen for disordered eating attitudes and behaviors characteristic of anorexia and bulimia nervosa. It includes 26 items rated on a 6-point Likert scale, with scoring weights assigned to each response and one reverse-coded item. Items form three subscales: Dieting, Bulimia and Food Preoccupation, and Oral Control. Internal consistency in the present study was acceptable to good: Dieting (α = 0.87), Bulimia and Food Preoccupation (α = 0.76), Oral Control (α = 0.66), and Composite Score (α = 0.87).

#### 2.2.5 Demographic and identity questionnaire

Participants reported age, education level, residential background (urban/rural), gender identity, sex assigned at birth, intersex status, hormone treatment, and sexual orientation. Definitions for terms such as “cisgender,” “transgender,” and “non-binary” were provided. Participants could select predefined categories or describe their identity and orientation in their own words.

#### 2.2.6 Optional feedback section

An open-ended feedback section was included at the end of the survey. Of the 22 responses, 13 expressed positive or appreciative sentiments, while 9 provided constructive suggestions. These included comments on item wording, representation of muscular or athletic body ideals, and cultural variability in gender expression. One participant highlighted challenges faced by sexual and gender minority (SGM) individuals in medical settings, an observation discussed further in the study’s limitations.

### 2.3 Data analysis

The study employed a cross-sectional observational design, with data collected at a single point in time without manipulation of variables. As several variables violated the assumption of normality, non-parametric and resampling-based methods were used for statistical analysis. Specifically, Mann–Whitney U tests were conducted to compare groups, and mediation analyses were performed using the percentile bootstrap method. For the planned mediation analyses using percentile bootstrap methods, prior literature was consulted. According to Fritz and MacKinnon (2007), a minimum of 558 participants is required to detect a small mediation effect (*a* = *b* = 0.14) with 80% power, while 71 participants are sufficient to detect medium effects (*a* = *b* = 0.39). The final sample included 270 participants, of whom 225 were SGM individuals, providing sufficient power to detect medium-to-large effects in the mediation models (Table 1).

**TABLE 1:** Empirical estimation of required sample size for percentile bootstrap mediation analysis.

Condition															
SS	SH	SM	SL	HS	HH	HM	HL	MS	MH	MM	ML	LS	LH	LM	LL
558	412	406	398	414	162	126	122	404	124	78	59	401	123	59	36

All sample sizes have been rounded up to the nearest whole number. Regarding the condition labels, the first letter refers to the size of path a, and the second letter refers to the size of path b; S = 0.14, H = 0.26, M = 0.39, L = 0.59.

## 3 Results

**FIGURE 1:**
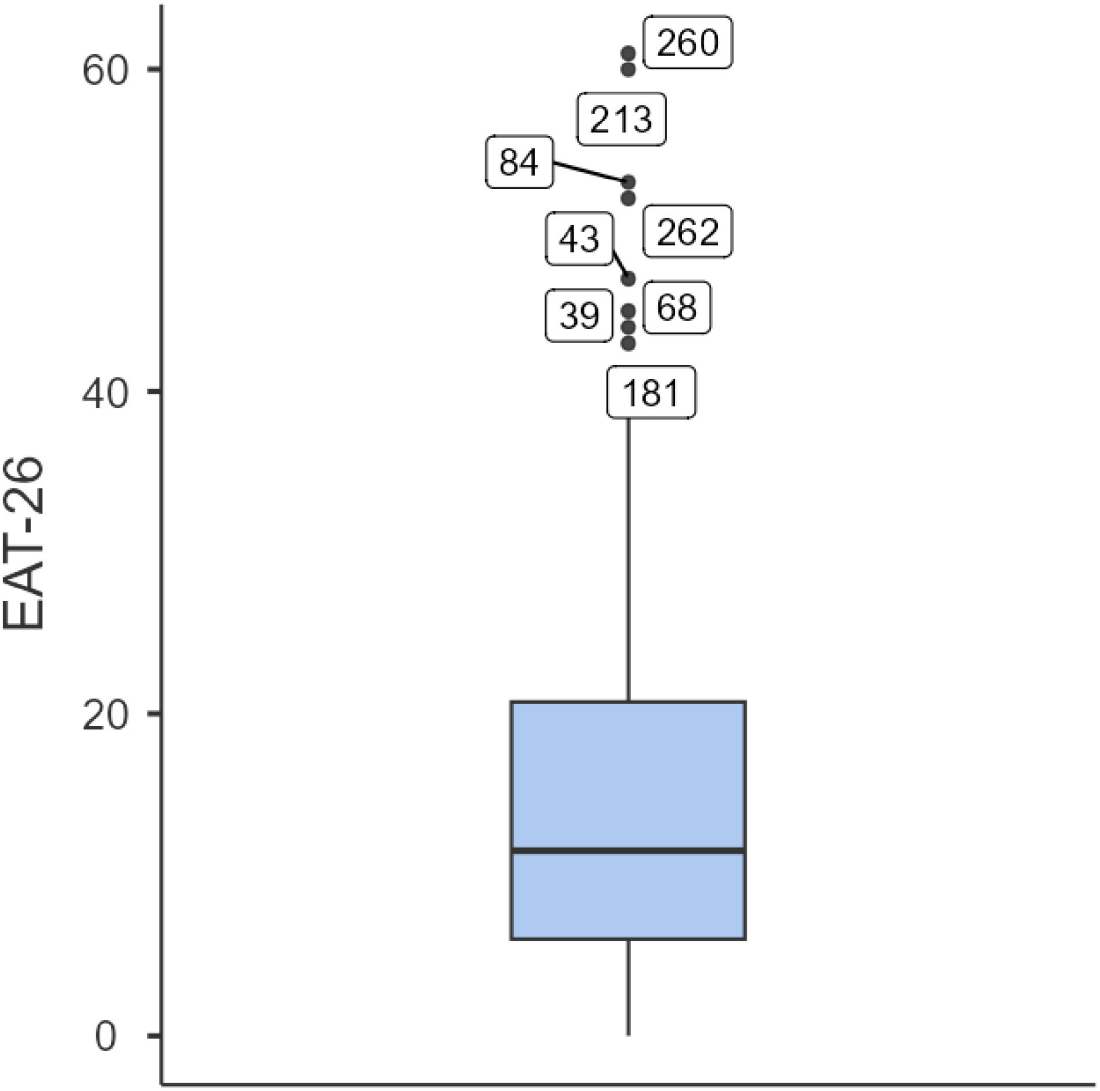
Outlier values for the eating disorders variable (EAT-26) (*N* = 270).

**FIGURE 2:**
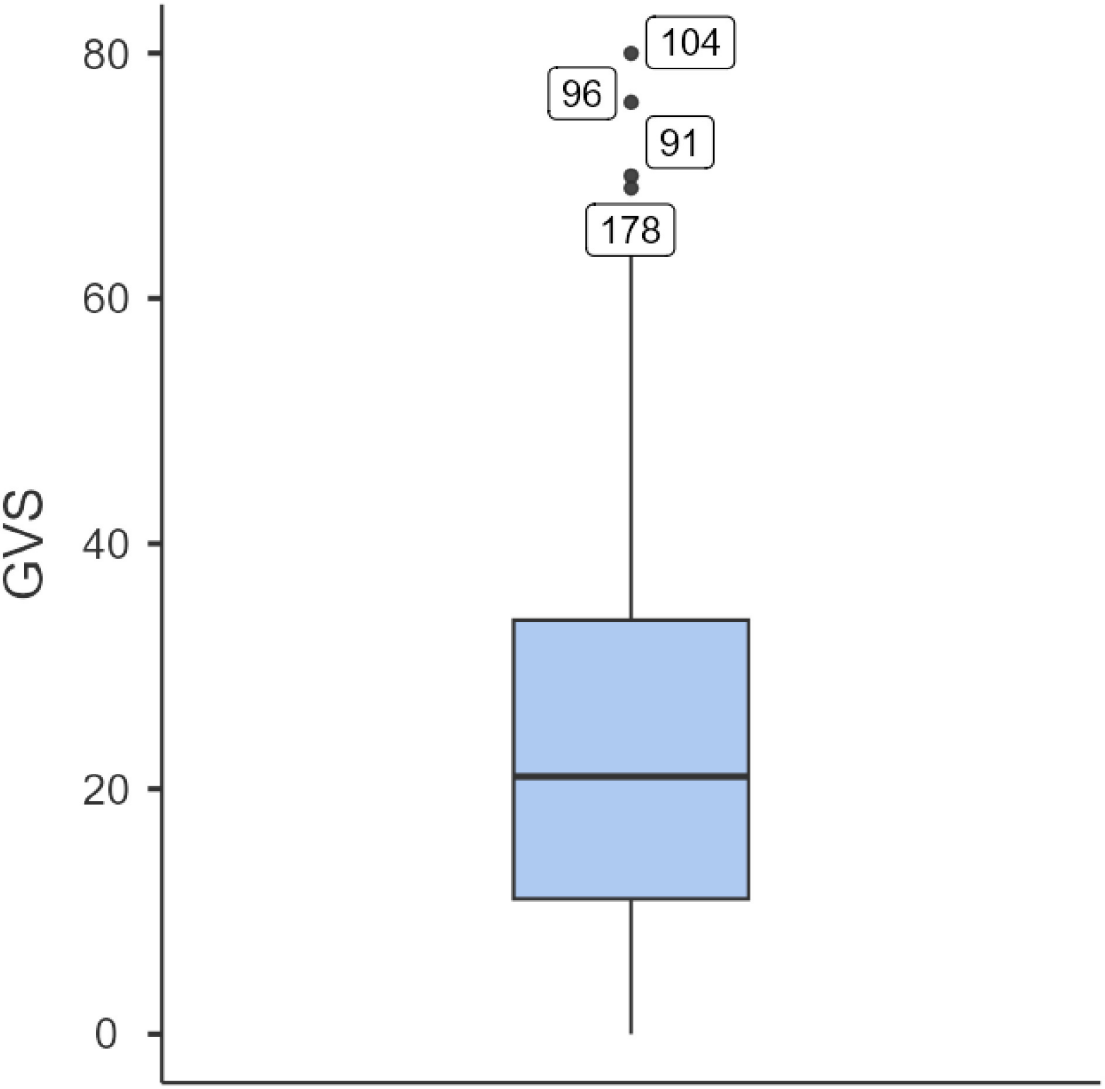
Outlier values for the gender variance variable (GVS) (*N* = 270).

No missing data were detected in the dataset. Box plot analysis was used to identify potential outliers. Eight outliers were found for the disordered eating variable (EAT-26; see Figure 1), and four for gender variance (GVS; see Figure 2). These values were retained in the analyses, as they were consistent with the study’s objectives. Such responses may reflect clinically relevant levels of eating pathology or high incongruence between sex assigned at birth and gender identity, which are characteristic of gender minority individuals. Table 2 displays the descriptive statistics for the full sample, while Table 3 presents the corresponding data for the SGM subgroup.

**TABLE 2:** Descriptive statistics for scalar variables in the total sample (*N* = 270).

Descriptive statistics	Femininity	EAT-26	GVS	SAAS	THIINA
N	270	270	270	270	270
Missing values	0	0	0	0	0
Mean	27.8	14.3	23.5	43.2	48.1
Median	29.0	11.5	21.0	40.5	48.0
Standard deviation	12.2	11.9	16.8	18.4	17.6
Minimum	5	0	0	16.0	17.0
Maximum	45	61	80.0	80.0	85.0
Skewness	−0.326	1.28	0.762	0.292	0.0879
ES skewness	0.148	0.148	0.148	0.148	0.148
Kurtosis	−1.15	1.51	0.200	−1.03	−0.970
ES kurtosis	0.295	0.295	0.295	0.295	0.295

**TABLE 3:** Descriptive statistics for scalar variables in the SGM group.

Descriptive statistics	Femininity	EAT-26	GVS	SAAS	THIINA
N	225	225	225	225	225
Missing values	0	0	0	0	0
Mean	28.3	15	26.2	45.7	48.7
Median	28.0	12	23.0	44.0	49.0
Standard deviation	11.2	12.1	16.5	18.2	18.2
Minimum	5.0	0	0	16.0	17.0
Maximum	45.0	61	80.0	80.0	85.0
Skewness	−0.277	1.27	0.706	0.139	0.0181
ES skewness	0.162	0.162	0.162	0.162	0.162
Kurtosis	−1.12	1.47	0.132	−1.07	−1.04
ES kurtosis	0.323	0.323	0.323	0.323	0.323
Shapiro-Wilk W	0.946	0.888	0.957	0.960	0.967
Shapiro-Wilk p	<0.001	<0.001	<0.001	<0.001	<0.001

### 3.1 Hypothesis 1

Given the violation of the normality assumption for EAT-26 scores (Shapiro–Wilk W = 0.890, *p* < 0.001), the non-parametric Mann–Whitney U test was used to compare disordered eating levels between the SGM and non-SGM groups. A one-tailed test was conducted. Results showed that EAT-26 scores were significantly lower in the non-SGM group (Median = 8) compared to the SGM group (Median = 12), *U* = 4007, *p* = 0.014 (one-tailed), 95% CI [−∞, −1.00]. This result supports the research hypothesis, suggesting that disordered eating behaviors tend to be more elevated among SGM individuals compared to non-SGM individuals.

To assess the preliminary relationships among key variables within the SGM group, bivariate correlations were computed (Table 4). As expected, disordered eating (EAT-26) showed strong, statistically significant positive correlations with both social appearance anxiety (SAAS; *r* = 0.54, *p* < 0.001) and thin-ideal internalization (THIINA; *r* = 0.66, *p* < 0.001), supporting their role as potential mediators. SAAS and THIINA were also positively correlated (*r* = 0.55, *p* < 0.001). Gender variance (GVS) was weakly and positively correlated with SAAS (*r* = 0.18), but this association did not reach the conservative significance threshold of *p* < 0.001. Similarly, Femininity was weakly correlated with THIINA (*r* = 0.13) and with EAT-26 (*r* = 0.17), though neither correlation achieved statistical significance. Overall, these patterns provide preliminary support for the hypothesized indirect pathways, while underscoring the need for further testing through mediation analyses.

**TABLE 4:** Pearson correlations among key variables in the SGM sample.

Variable	EAT	SAAS	THIINA	GVS	Femininity
1. Disordered eating (EAT-26)	–				
2. Social appearance anxiety (SAAS)	0.54*	–			
3. Thin-ideal internalization (THIINA)	0.66*	0.55*	–		
4. Gender variance (GVS)	−0.04	0.18	−0.02	–	
5. Femininity	0.17	−0.02	0.13	−0.48*	–

*Correlation is significant at the *p* < 0.001 level.

### 3.2 Hypothesis 2

**FIGURE 3:**
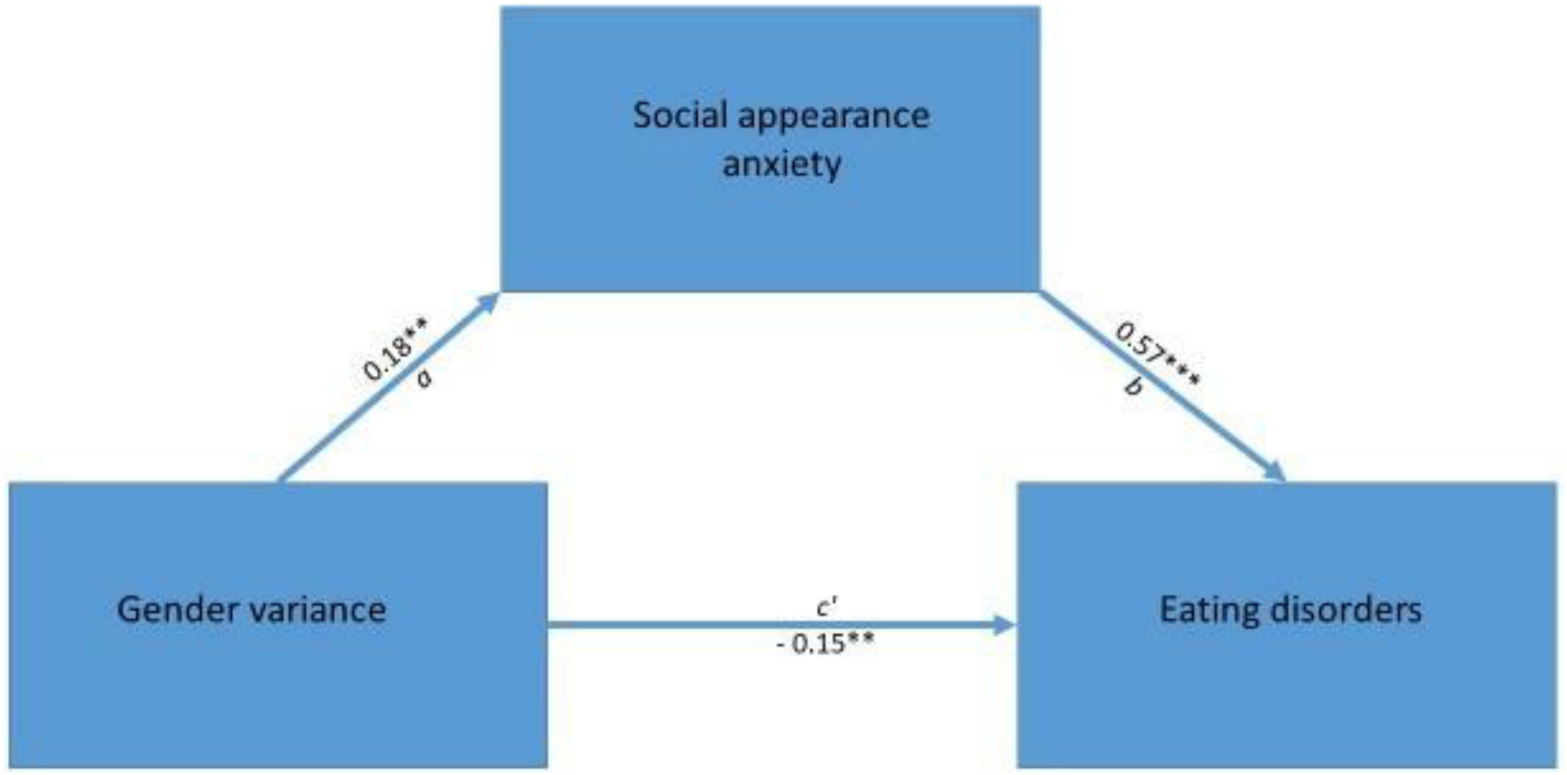
Mediation effect of appearance-related social anxiety (SAAS). The symbols indicate significance levels: **p < 0.01, ***p < 0.001.

A percentile bootstrap mediation analysis was conducted to test whether social appearance anxiety (SAAS) mediated the relationship between gender variance (GVS) and disordered eating (EAT-26) among SGM participants (see Figure 3). As shown in Table 5, the total effect of gender variance on disordered eating was not statistically significant (β = −0.0428, *z* = −0.642, *p* = 0.521, 95% CI [−0.1301, 0.0656]). However, the direct effect of gender variance on disordered eating, in the absence of the mediator, was significant and negative (β = −0.1465, *z* = −2.615, *p* = 0.009, 95% CI [−0.1981, −0.0140]). The indirect effect through social appearance anxiety was significant and positive (β = 0.1036, *z* = 2.670, *p* = 0.008, 95% CI [0.0101, 0.1325]); the confidence interval for this effect did not include zero, indicating a statistically significant mediation. Gender variance was significantly associated with social appearance anxiety, although the effect was small (β = 0.1813, *z* = 2.766, *p* = 0.006, 95% CI [0.0258, 0.3462]). In turn, social appearance anxiety was strongly associated with disordered eating (β = 0.5715, *z* = 10.206, *p* < 0.001, 95% CI [0.3001, 0.4641]). These findings indicate a pattern of associations consistent with partial mediation, with the indirect and direct effects operating in opposite directions, effectively canceling each other out in the total effect.

**TABLE 5:** Mediation analysis: appearance-related social anxiety as a mediator (*N* = 225).

CI 95%							
	Estimated	*SE*	*LL*	*UL*	β	z	p
Indirect	0.0761	0.0285	0.0101	0.1325	0.1036	2.670	0.008
Path a	0.1990	0.0720	0.0258	0.3462	0.1813	2.766	0.006
Path b	0.3823	0.0375	0.3001	0.4641	0.5715	10.206	<0.001
Direct	−0.1075	0.0411	−0.1981	−0.0140	−0.1465	−2.615	0.009
Total	−0.0314	0.0490	−0.1301	0.0656	−0.0428	−0.642	0.521

SE denotes the standard error of the estimate; CI denotes the confidence interval calculated using the bootstrap-percentile method; LL denotes the lower limit; UL denotes the upper limit; β denotes the completely standardized effect size.

### 3.3 Hypothesis 3

**FIGURE 4:**
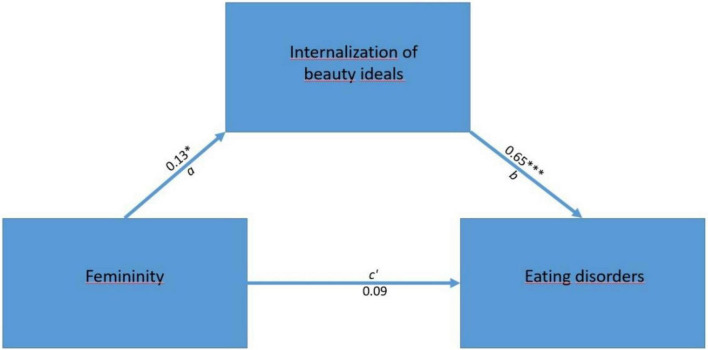
Mediation effect of internalization of beauty ideals (THIINA). The symbols indicate significance levels: *p < 0.05, ***p < 0.001.

Another percentile bootstrap mediation analysis was conducted to examine whether thin ideal internalization (THIINA) mediated the relationship between femininity and disordered eating (EAT-26) among SGM participants (see Figure 4). As shown in Table 6, the total effect of femininity on disordered eating was statistically significant (β = 0.1709, *z* = 2.60, *p* = 0.009, 95% CI [0.0334, 0.3480]). However, the direct effect was not significant (β = 0.0856, *z* = 1.72, *p* = 0.086, 95% CI [−0.0295, 0.2110]), and the indirect effect via thin ideal internalization approached the threshold for significance (β = 0.0852, *z* = 1.95, *p* = 0.051, 95% CI [−0.0094, 0.2020]). Thin ideal internalization showed a strong positive association with disordered eating, with a large effect size (β = 0.6549, *z* = 13.14, *p* < 0.001, 95% CI [0.3641, 0.5080]). Femininity was a marginally significant predictor of thin ideal internalization, with a small effect size (β = 0.1302, *z* = 1.97, *p* = 0.049), although the corresponding confidence interval included zero (95% CI [−0.0219, 0.4400]).

**TABLE 6:** Mediation analysis: internalization of beauty ideals as a mediator (*N* = 225).

CI 95%							
	Estimated	*SE*	*LL*	*UL*	β	z	p
Indirect	0.0927	0.0476	−0.00941	0.202	0.0852	1.95	0.051
Path a	0.2120	0.1076	−0.02185	0.440	0.1302	1.97	0.049
Path b	0.4375	0.0333	0.36405	0.508	0.6549	13.14	<0.001
Direct	0.0932	0.0542	−0.02948	0.211	0.0856	1.72	0.086
Total	0.1859	0.0716	0.03337	0.348	0.1709	2.60	0.009

SE represents the standard error of the estimate; CI represents the confidence interval calculated using the bootstrap-percentile method; LL denotes the lower limit; UL denotes the upper limit; β denotes the fully standardized effect size.

## 4 Discussion

This study extends existing research by examining sexual and gender minority (SGM) individuals in Romania, an Eastern European context largely underrepresented in the literature on eating disorders. Cultural, social, and legislative differences across countries may shape body image concerns and disordered eating differently, highlighting the importance of context-specific investigations.

One of the main objectives of this study was to compare disordered eating behaviors between sexual and gender minority (SGM) individuals and their cisgender heterosexual counterparts. Prior research has consistently shown that SGM individuals are at greater risk for body image concerns and eating pathology (Parker and Harriger, 2020), with higher prevalence rates of diagnoses such as binge eating disorder, anorexia nervosa, and bulimia nervosa (Nagata et al., 2018, Valenta et al., 2024), as well as more severe symptoms at the time of treatment admission (Mensinger et al., 2020). The present findings are consistent with this literature, showing that SGM participants reported higher levels of disordered eating than cisgender heterosexual individuals.

Beyond these group differences, the study also sought to explore associative patterns that could account for differences in disordered eating behaviors among SGM individuals. Regarding the mediation effect of social appearance anxiety, the results were statistically significant. Specifically, social appearance anxiety mediated the relationship between gender variance and disordered eating, such that higher gender variance was associated with greater appearance-related anxiety, which in turn was positively associated with disordered eating levels. This finding may suggest that individuals whose gender identity or expression deviates from socially normative expectations may experience heightened anxiety about being socially rejected based on appearance. As a result, they may become hypervigilant about their bodies, place greater importance on appearance ideals, or become more vulnerable to self-objectification. Transgender individuals, in particular, may experience anxiety related to whether their physical appearance is perceived as congruent with their gender identity (commonly referred to as “passing”). In such cases, some disordered eating behaviors might be perceived by individuals as a way to alter secondary sex characteristics in order to align one’s body with one’s gender identity. This effect may be exacerbated by limited access to gender-affirming medical procedures.

Interestingly, despite the indirect pathway indicating increased disordered eating via appearance-related anxiety, the direct effect of gender variance was negative. This suggests that for some individuals, greater gender variance may signal psychological flexibility or resilience, perhaps through a more integrated gender identity (Radu, 2017). These contrasting effects highlight the complexity of gender expression as both a potential stressor and a protective factor, depending on individual and contextual variables.

In the mediation analysis involving thin ideal internalization, the direct effect of femininity on disordered eating was not statistically significant. Femininity marginally predicted internalization of thinness ideals, with a small effect size and a confidence interval that included zero. The indirect effect of femininity on disordered eating, via thin ideal internalization, was also non-significant but approached the conventional threshold for significance. This borderline result could reflect limited statistical power, increasing the likelihood of a Type II error. According to MacKinnon (2008), detecting an indirect effect with a small path a (≈ 0.14) and a large path b (≈ 0.59) requires a minimum sample size of 398 participants to achieve 80% power. Given that the current mediation model was tested on 225 SGM participants, the marginal result may partly reflect insufficient statistical power rather than a true absence of effect. Nevertheless, the pattern of results aligns with prior findings. For instance, Green et al. (2008) reported no relationship between disordered eating and adherence to traditional femininity norms, except in domains explicitly related to appearance. Moreover, in some transgender individuals, in some cases, the drive to lose weight may be linked to a desire to suppress secondary female sex characteristics such as breast or hip size (Ålgars et al., 2012).

It is also plausible that evolving beauty standards, particularly those shaped by social media content, may be linked to changes in the way disordered eating is expressed in recent years. While most major platforms now enforce strict policies against explicit promotion of anorexia or bulimia, a shift has occurred from the overtly restrictive “heroin chic” aesthetic of the late 20th century to a more subtle “healthy lifestyle” ideal. Despite appearing more benign, this ideal may continue to mirror or be associated with core features of disordered eating, such as perfectionism, compulsive control, body dissatisfaction, and compensatory behaviors, under the guise of wellness. Recent research suggests that exposure to “slim-thick” ideals (i.e., bodies that are simultaneously thin and muscular) may be associated with even more negative body image outcomes than thinness alone (McComb and Mills, 2021), especially among populations navigating complex gendered appearance norms.

The unique cultural and structural conditions of Romania may both contribute to and intensify these associations. Romania remains one of the most socially conservative countries in the European Union, with low public support for LGBTQ+ rights and limited institutional recognition or protection for sexual and gender minorities. Persistent stigma, fear of rejection, and lack of access to gender-affirming care may increase body-related anxiety and internalized appearance pressures among SGM individuals (Bochicchio et al., 2025).

Additionally, the influence of traditional gender norms, rooted in patriarchal values, rigid binary expectations, and reinforced by dominant religious discourses (Miscoiu et al., 2022) may constrain the ways in which individuals can express gender variance without experiencing social or psychological costs. These cultural pressures can reinforce the link between appearance-based anxiety and disordered eating, especially in environments where deviations from gender norms are met with ridicule, discrimination, or social exclusion. The lack of inclusive mental health education and support services further compounds these vulnerabilities by limiting access to affirming care and early intervention.

While this study provides valuable insights into disordered eating behaviors among SGM individuals in Romania, the findings should be interpreted with caution. The sample was not representative of the broader LGBTQ+ population or the Romanian SGM community as a whole. Therefore, results cannot be generalized beyond the specific characteristics of this sample.

### 4.1 Limitations

One key limitation of this study lies in the use of the EAT-26, an instrument not specifically designed or validated for diverse populations such as sexual and gender minorities (SGM). Like many legacy measures for eating disorders, the EAT-26 was developed to assess the symptomatology of cisgender heterosexual women from higher socioeconomic backgrounds. As a result, it may not fully capture the diverse expressions of disordered eating among SGM individuals. For instance, the measure tends to prioritize weight loss as a core indicator, which may not encompass other motivations such as body masculinization, gender congruence, or increased muscularity.

Moreover, the EAT-26 does not assess compensatory behaviors such as laxative use, diet pill consumption, or substance use, behaviors that may be particularly salient among SGM populations. Some research (Marshal et al., 2008; Warren et al., 2012; Demant et al., 2016; Hill et al., 2022) has highlighted that individuals in these groups are more likely to engage in substance use for appetite suppression and body control, including energy drinks, smoking, or appearance-and performance-enhancing drugs (APEDS). These behaviors may co-occur with or be shaped by experiences of gender dysphoria or sociocultural pressures around body presentation, yet remain unmeasured by conventional instruments.

Relatedly, the present study did not assess muscle dysmorphia, which may be highly relevant in LGBTQ+ populations. Although the EAT-26 and THIINA address thinness-oriented concerns, they fail to account for muscularity-driven body image disturbances. Muscle dysmorphia, is conceptually linked to eating disorders through shared features such as perfectionism, compulsive exercise, and emotional avoidance. Emerging literature suggests this construct is particularly salient among gay and bisexual men and may be underrecognized in standard assessments (Fabris et al., 2020).

Although the study was conducted in Romania, where the majority of the population identifies as ethnically Romanian, the lack of data on participants’ ethnic backgrounds remains a limitation. This omission precludes a more nuanced intersectional analysis, particularly with respect to minority ethnic groups such as Roma individuals, who may face additional layers of marginalization. Likewise, although educational attainment was recorded, no indicators of income or occupational status were collected. Given the established links between food insecurity, financial strain, and disordered eating–especially among LGBTQ+ populations (Choi et al., 2019)–the absence of detailed socioeconomic data is a notable constraint.

Language and cultural adaptation posed further challenges. Feedback from participants highlighted concerns with the Romanian translation of key terms, such as the rendering of thin in the THIINA scale. While subţire was used to avoid connotations of weakness (slab) or athleticism (suplu), some respondents found it vague or unfamiliar. These nuances might have impacted the way items were interpreted, potentially affecting response validity.

The sample’s demographic composition also limits the generalizability of findings. In both SGM and non-SGM groups, cisgender women were overrepresented, and the distribution of SGM subgroups was uneven. The majority of SGM participants were assigned female at birth, and the most commonly reported gender identity was cisgender woman. This skew is consistent with known biases in online and LGBTQ+ -friendly recruitment channels. As such, findings may disproportionately reflect the experiences of certain subgroups while underrepresenting others, such as transgender men or non-binary individuals with low visibility.

Finally, the statistical power of the mediation analyses may have been insufficient to detect small indirect effects. While 225 SGM participants were included in these models, this number falls short of sample sizes typically required to detect subtle mediation effects–especially when the independent variable exerts a weak influence on the mediator but the mediator strongly predicts the outcome. Non-significant or marginally significant mediation effects, such as those involving thin-ideal internalization, may therefore be influenced by limited power, raising caution about interpreting these results as a lack of association. Nevertheless, observed effect sizes suggest underlying patterns that warrant further investigation.

### 4.2 Future research directions

Future research should adopt intersectional and longitudinal designs to clarify how gender identity, body image concerns, and disordered eating interact over time, particularly within marginalized populations. In Eastern European contexts, such as Romania, where LGBTQ+ individuals face unique cultural, political, and social challenges, these dynamics may unfold differently than in Western societies. The mechanisms linking gender variance, appearance anxiety, and eating pathology remain insufficiently understood, and context-specific factors such as stigma, identity integration, and access to gender-affirming care must be more thoroughly investigated.

To address current measurement gaps, future research should consider using or adapting assessment tools that have been previously applied in studies with sexual and gender minority (SGM) populations, even if not originally developed for these groups. These instruments should move beyond thinness-focused models to capture a broader range of appearance-related concerns, such as muscularity, gender congruence, and the use of appearance- and performance-enhancing substances. For example, the Eating Disorder Examination Questionnaire (EDE-Q) has been employed in studies involving LGBTQ+ participants (Nagata et al., 2019) and may offer a more nuanced assessment than the EAT-26. Additionally, constructs like muscle dysmorphia and gender-specific drives for body modification, particularly relevant for transgender and non-binary individuals, could be better assessed using tools such as the Muscle Dysmorphic Disorder Inventory (MDDI; Zeeck et al., 2018). Incorporating such instruments may help capture the diverse experiences of SGM individuals more accurately.

Recruitment strategies should aim for more balanced and representative samples. Stratified or randomized sampling across diverse geographic, socioeconomic, and gender identity groups would enhance generalizability. Future studies should actively include underrepresented subpopulations, such as transgender men, rural SGM individuals, ethnic minorities, or those with lower levels of identity disclosure, who are often excluded from convenience samples.

Finally, evolving beauty standards, particularly those amplified by social media, warrant deeper investigation. Future studies should explore how platform-specific content (e.g., Instagram’s fitness culture vs. TikTok’s body positivity trends) interacts with gender identity and internalized appearance ideals to shape eating behaviors. Qualitative and mixed-methods approaches could provide richer insight into lived experiences and support the development of culturally responsive, inclusive prevention and intervention strategies tailored to Eastern European SGM populations.

## 5 Conclusion

This study contributes to a growing body of evidence showing that sexual and gender minority (SGM) individuals face elevated risk for disordered eating behaviors compared to cisgender heterosexual individuals. In addition to confirming previously observed group differences, the study explored underlying psychosocial mechanisms, identifying social appearance anxiety as a significant partial mediator of the relationship between gender variance and disordered eating. These findings highlight the role of appearance-related anxiety in individuals whose gender expression diverges from societal expectations, an effect that may be intensified in conservative cultural contexts such as Romania.

Although the internalization of thinness ideals did not significantly mediate the relationship between femininity and disordered eating, the effect approached significance and may warrant further investigation, particularly in light of evolving beauty standards shaped by social media and community norms. The unexpected negative direct effect of gender variance on eating pathology raises the possibility that greater gender flexibility may, in some cases, serve as a psychological resource, supporting identity coherence and body acceptance.

Overall, these findings underscore the importance of developing culturally sensitive assessment tools and interventions that reflect the lived experiences of SGM individuals in Eastern Europe. Future research should further examine the complex interplay between gender identity, sociocultural pressures, and disordered eating behaviors, including culturally embedded constructs such as muscularity-driven dissatisfaction, compensatory behaviors, and intersecting minority stressors.

## Data Availability

The raw data supporting the conclusions of this article will be made available by the authors, without undue reservation.
